# Effects of early side-alternating whole-body vibration training on neuromuscular control and function after ACL reconstruction: a randomized controlled trial

**DOI:** 10.3389/fspor.2026.1799820

**Published:** 2026-07-02

**Authors:** Yiwei Wang, Jingfeng Ma, Zhibin Huang, Zhijiao Fan, Xinran Li, Wanting Li, Yubao Ma

**Affiliations:** 1 Musculoskeletal Rehabilitation Center of Beijing Rehabilitation Hospital, Capital Medical University, Beijing, China; 2 College of Exercise and Health, Shenyang Sport University, Shenyang, China; 3 School of Physical Education and Exercise Rehabilitation, Jinzhou Medical University, Jinzhou, China; 4 Rehabilitation Treatment Center, Beijing Rehabilitation Hospital, Capital Medical University, Beijing, China

**Keywords:** anterior cruciate ligament reconstruction, isokinetic, knee joint, proprioception, rehabilitation, side-alternating vibration

## Abstract

**Objective:**

This study aims to evaluate the rehabilitation efficacy of side-alternating whole-body vibration training (SAV-WBVT) combined with conventional rehabilitation training for male patients undergoing anterior cruciate ligament reconstruction (ACLR).

**Methods:**

Between January 2024 and March 2025, thirty-four male patients (0–4 weeks post-ACLR) were initially recruited and randomly allocated to a control group (*n* = 17) or a whole-body vibration training (WBVT) group (*n* = 17). During the 8-week trial, 6 patients dropped out, leaving 14 patients per group for the final analysis. Both groups received conventional rehabilitation training, while the WBVT group concurrently received SAV-WBVT. Intra- and inter-group comparisons were evaluated pre-intervention and post-8-week intervention using two-way repeated measures ANOVA.

**Results:**

All indicators significantly improved over time in both groups. Specifically, compared to the control group, the WBVT group demonstrated significantly superior outcomes at the post-8-week intervention assessment in knee extensor and flexor peak torque, joint position sense (90°–60°, 90°–30°), static balance (eyes open and closed), and Lysholm Knee Scores. However, there was no significant between-group difference in joint kinesthesia improvement.

**Conclusions:**

Early rehabilitation after ACLR has a significant time effect, and the 8-week SAV-WBVT combined with conventional rehabilitation program starting at 5 weeks after surgery is superior to conventional rehabilitation training alone in improving muscle strength, joint position sense, static balance, and patients' subjective functional perception. It is recommended to include it in the early rehabilitation program after ACLR.

**Clinical Trial Registration:**

https://www.chictr.org.cn/showproj.html?proj=215786, ChiCTR2400079359.

## 1 Introduction

Anterior cruciate ligament (ACL) rupture is a common sports injury often leading to long-term sequelae, including muscle weakness, proprioceptive deficits, functional impairment, and knee osteoarthritis (1). Anterior cruciate ligament reconstruction (ACLR) is the mainstream treatment for ACL rupture, serving as the standard of care to restore knee stability and facilitate return to sport for young, active populations (2).

Despite continuous advancements in surgical techniques, graft selection, and rehabilitation, outcomes remain suboptimal. Indeed, a substantial proportion of patients face persistent challenges in regaining their pre-injury athletic performance and remain highly susceptible to long-term joint degeneration (3). Early post-ACLR recovery is frequently characterized by quadriceps atrophy, strength deficits, knee pain, and restricted joint mobility. This phenomenon is primarily attributed to arthrogenic muscle inhibition (AMI) caused by factors such as postoperative swelling, pain, joint laxity, and inflammation (4). Conventional rehabilitation strategies typically rely on voluntary quadriceps engagement. However, this approach is often hindered by ongoing AMI, which diminishes the accessible motor neuron pool and intrinsically restricts the patient's capacity for voluntary recruitment (5). Consequently, effective interventions addressing this neurogenic dysfunction are urgently required.

As a novel rehabilitative strategy, whole-body vibration training (WBVT) has garnered significant attention for postoperative ACL care. This intervention utilizes a vibrating platform to deliver mechanical stimuli to the body, stimulating the muscle-tendon complex to elicit reflexive contractions known as the tonic vibration reflex (TVR), thereby driving subsequent adaptive physiological changes (6). In experimental models of effusion-induced quadriceps dysfunction, WBVT has been demonstrated to significantly enhance peak torque and voluntary activation (7). These benefits are mediated by the TVR, a mechanism wherein mechanical oscillations excite muscle spindles, subsequently activating alpha motor neurons to elicit involuntary contractions (8).

Common WBVT platforms are categorized into synchronous vertical vibration (SVV) and side-alternating vibration (SAV). SVV platforms oscillate the entire plate vertically to generate uniform sinusoidal stimulation. In contrast, SAV platforms utilize a fulcrum-based mechanism to deliver asynchronous vibration by alternately raising and lowering the left and right sides (9). This distinct mechanism creates differential vertical displacement, promoting rotational movement in the hip and lumbosacral regions. This action passively stretches the muscle-tendon complex, thereby enhancing neuromuscular activation. Clinically, SVV offers a safety advantage post-ACLR by minimizing relative tibiofemoral motion, whereas SAV demonstrates superior efficacy in enhancing quadriceps activation (10).

Previous studies have explored the incorporation of WBV into exercise therapy following ACL reconstruction, demonstrating broad potential in neuromuscular recovery. Recent literature indicates that WBV combined with exercise therapy can significantly enhance the corticomotor excitability of the quadriceps in athletes post-ACLR, addressing central neural inhibition (11). Furthermore, randomized controlled trials have reported that such combinations improve dynamic functional outcomes, including jump-landing stability and dynamic postural control (12).

Despite these findings, the existing literature presents conflicting results regarding the optimal timing, specific vibration parameters, and the exact trajectory of sensory recovery. While previous studies have primarily evaluated synchronous vertical vibration or initiated interventions in the later phases of rehabilitation, a specific knowledge gap remains regarding the efficacy of early intervention. Therefore, the novelty of the current study lies in being one of the first to apply a SAV protocol in the early postoperative phase (starting at 5 weeks post-ACLR). Unlike traditional platforms, SAV generates differential vertical displacements that actively mimic human gait kinetics, hypothetically offering superior involuntary muscle activation and proprioceptive recalibration when AMI is most profound.

Therefore, this study aims to evaluate the clinical efficacy of a novel rehabilitation strategy: Side-Alternating Vibration (SAV-WBVT). We hypothesized that the early incorporation of SAV-WBVT (initiated 5 weeks postoperatively) would yield superior improvements in knee muscle strength, proprioceptive recalibration, and static balance compared to conventional rehabilitation alone. By ameliorating acute quadriceps dysfunction, this approach holds promise for accelerating short-term functional recovery and improving patient compliance.

## 2 Materials and methods

### 2.1 Inclusion and exclusion criteria

Inclusion Criteria: (1) Male patients aged 18–40 years. (2) Unilateral ACL rupture. (3) Undergoing primary ACLR with an autologous hamstring tendon graft. (4) 0–4 weeks postoperatively. (5) Patients without other orthopedic diseases or previous history of other conditions on their lower limbs.

Exclusion Criteria: (1) Previous injury and/or surgery to the ipsilateral or contralateral lower limb. (2) Having undergone meniscal repair. (3) Combined injuries of the collateral ligaments, posterior cruciate ligament, or joint dislocation. (4) Active knee flexion range of motion (ROM) <90° by the 5th postoperative week. (5) Contraindications to WBVT: Acute thrombosis or acute arterial occlusion, acute inflammation of the musculoskeletal system, acute disc herniation and abdominal hernia, recent fractures, kidney stones and gallstones, unhealed wounds, rheumatoid arthritis, epilepsy. (6) Previous exposure to WBVT, to avoid training or memory effects (Table 1).

**Table 1:** Basic characteristics of participants (mean ± SD).

Item	Control group (*n* = 14)	WBVT group (*n* = 14)	Statistic (t/χ^2^)	*P*
Age (years)	31.00 ± 4.19	31.14 ± 4.07	0.091	0.928
Height (cm)	175.50 ± 3.82	177.14 ± 5.08	0.967	0.342
Weight (kg)	70.07 ± 4.14	72.50 ± 4.62	1.465	0.155
BMI (kg/m^2^)	22.74 ± 0.76	23.12 ± 0.70	1.420	0.167
Affected side (left/right)	4/10	3/11		>0.99

Data are presented as mean ± SD. For “Affected Side”, data represent the count of Left/Right. *P*-values were derived using the independent samples *t*-test for continuous variables and the Chi-square test for categorical variables.

### 2.2 Sample size calculation

The sample size was estimated *a priori* using G*Power software (version 3.1.9.7). Based on a two-way repeated measures ANOVA (within-between interaction), with an anticipated effect size (*f*) of 0.25, an alpha level (*α*) of 0.05, and a statistical power of 0.80 across two groups and three measurement time points, a minimum total sample size of 28 participants was required. Accounting for an estimated 20% attrition rate during the trial, 34 participants were initially recruited for this study.

### 2.3 Ethics, randomization, and blinding

The study protocol obtained formal ethical approval from the Ethics Committee of Beijing Rehabilitation Hospital, Capital Medical University (Approval No. 2023bkky-090). Written informed consent was obtained from all participants prior to enrollment, in accordance with the Declaration of Helsinki.

Participants were randomly allocated (1:1 ratio) to either the Control or WBVT group using a computer-generated randomization sequence created by an independent researcher. Allocation concealment was ensured using sequentially numbered, opaque, sealed envelopes (SNOSE). Due to the physical nature of the WBVT intervention, it was not possible to blind the participants or the treating physical therapists. However, to minimize detection bias, all outcome assessments and statistical analyses were performed by independent assessors who were strictly blinded to the group allocation.

### 2.4 Experimental procedures

The 12-week rehabilitation program comprised 36 supervised sessions administered 3 times per week. The specific progression of conventional physical therapy, strength, and proprioceptive exercises across Phase 1 (t0), Phase 2 (t1), and Phase 3 (t2) is detailed in Table 2.

**Table 2:** Rehabilitation plans for each stage.

Category	Training items	Dosage/Description	Pre-intervention	Mid-intervention	Post-intervention
Physiotherapy	Low-frequency electrical stimulation	20 min, intensity based on comfort	●	●	
	Patellar mobilization	10 min, superior/inferior/lateral	●		
Muscle Strength (Basic)	Quadriceps isometric contraction	10 s hold, 10 reps/set, 3 sets	●		
	Active knee flexion (Sitting)	15 reps/set, 3 sets	●	●	
	Hip extension (Side-lying)	10 reps/set, 3 sets	●	●	
	Hip abduction (Side-lying)	10 reps/set, 3 sets	●	●	
Muscle Strength (Advanced)	Active knee extension (Sitting)	15 reps/set, 3 sets		● (90°–45°)	● (90°–0°)
	Step-up training	10 reps/set, 3 sets		●	
	Step-down training	10 reps/set, 3 sets			●
	Knee flexion (Prone)	15 reps/set, 3 sets, 3–5 s hold			●
	Resisted lateral walking	10 steps/set, 3 sets			●
Proprioception	Balance training	10 min, static to dynamic		●	
	Gait training	10 min, lateral crossover/accelerated			●
Intervention (Variable)	Squat (on SAV platform)	10 min (1 min/set, 1 min rest), 60° flexion		●	●

● indicates that the specific training item was performed during the respective rehabilitation phase. SAV: side-alternating vibration. WBVT: whole-body vibration training.

At week 5, participants achieving ≥90° active knee flexion commenced the 8-week experimental intervention (24 total WBVT sessions). The WBVT group performed barefoot static squats (60° knee flexion) on a side-alternating platform (wellengang, SVG Medizinsysteme GmbH, Germany). Each 10-minute session comprised five 1-minute vibration sets alternating with 1-minute rests. Vibration parameters progressed biweekly to ensure continuous adaptation, increasing from 11 to 13 Hz (2 mm amplitude) in weeks 5–6, to 14–16 Hz (2 mm) in weeks 7–8, 17–19 Hz (3 mm) in weeks 9–10, and finally 20 Hz (3 mm) in weeks 11–12. The control group performed identical squats on a deactivated platform. All concurrent rehabilitation volumes were strictly matched between cohorts.

### 2.5 Calculation of outcome variables

Outcome variables were quantified as follows:

Isokinetic Peak Torque (PT, N·m): Defined as the maximum concentric torque achieved at 60°/s, automatically extracted via the dynamometer's proprietary software.
Proprioceptive Absolute Error (AE, °): Calculated to eliminate directional bias. JPS AE was the absolute difference between target and actively reproduced angles (AE = |reproduced - target|); TTDPM AE was the absolute difference between initial and first perceived movement angles (AE = |detection - initial|).
Static Balance (COP Ellipse Area, mm^2^): Computed via the platform's integrated software using principal component analysis of anteroposterior and mediolateral sway trajectories, representing the area enclosing 95% of the COP data points.
Lysholm Knee Score: Calculated as the cumulative sum of its eight predefined functional domains (score range: 0–100).

### 2.6 Statistical analysis

To standardize the timeline and align it with the 8-week experimental intervention, all outcome assessments were categorized into three specific time points: pre-intervention (t0, at 5 weeks postoperatively), mid-intervention (t1, at 9 weeks postoperatively), and post-intervention (t2, at 13 weeks postoperatively).

Statistical analyses were performed using SPSS 27.0. Baseline characteristics were compared using independent samples *t*-tests for continuous variables (e.g., age, height, weight, BMI) and Chi-square tests for categorical variables (e.g., affected side). A two-way repeated measures ANOVA (Group × Time) was used to analyze muscle strength, proprioception, balance, and Lysholm scores. Simple effects analysis was conducted for significant interactions. Significance was set at *P* < 0.05. Prior to the ANOVA, the normality of data distribution was assessed using the Shapiro–Wilk test, and the homogeneity of variances was verified using Levene's test.

## 3 Results

### 3.1 Participant flow and baseline characteristics

**Figure 1:**
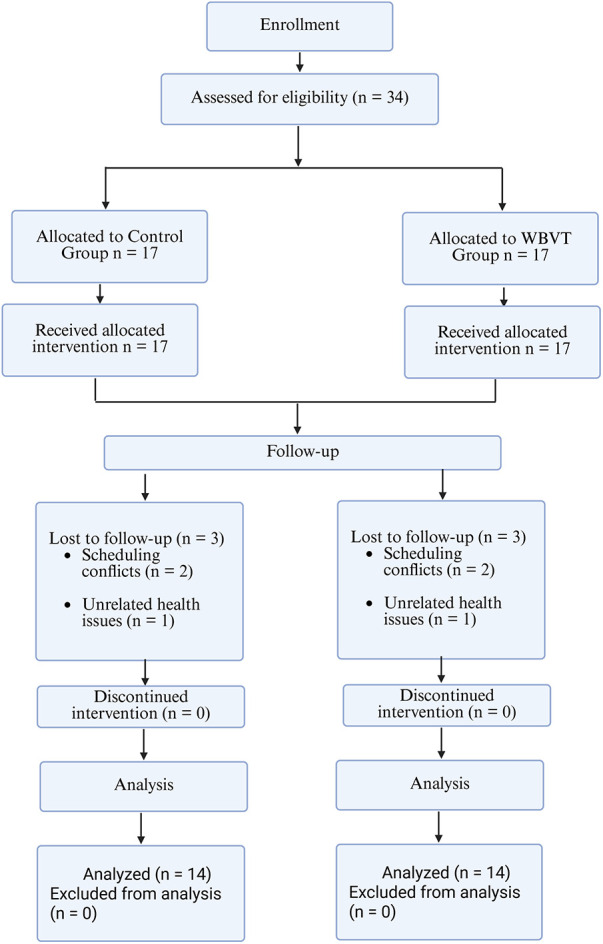
The flow of participants through each stage of the randomized controlled trial.

Between January 2024 and March 2025, 34 eligible patients (*n* = 34) were recruited and randomized in a 1:1 ratio to either the control group (*n* = 17) or the WBVT group (*n* = 17). During the 8-week intervention period, six participants withdrew from the study (two due to unrelated health issues and four due to scheduling conflicts). Specifically, three participants dropped out from the control group, and three from the WBVT group. Consequently, 28 participants (*n* = 14 per group) completed the intervention and follow-up assessments. Baseline demographic and clinical characteristics of the analyzed participants were comparable between groups (*P* > 0.05) (Figure 1).

### 3.2 Isokinetic muscle strength testing

#### 3.2.1 Knee extensor peak torque

**Figure 2:**
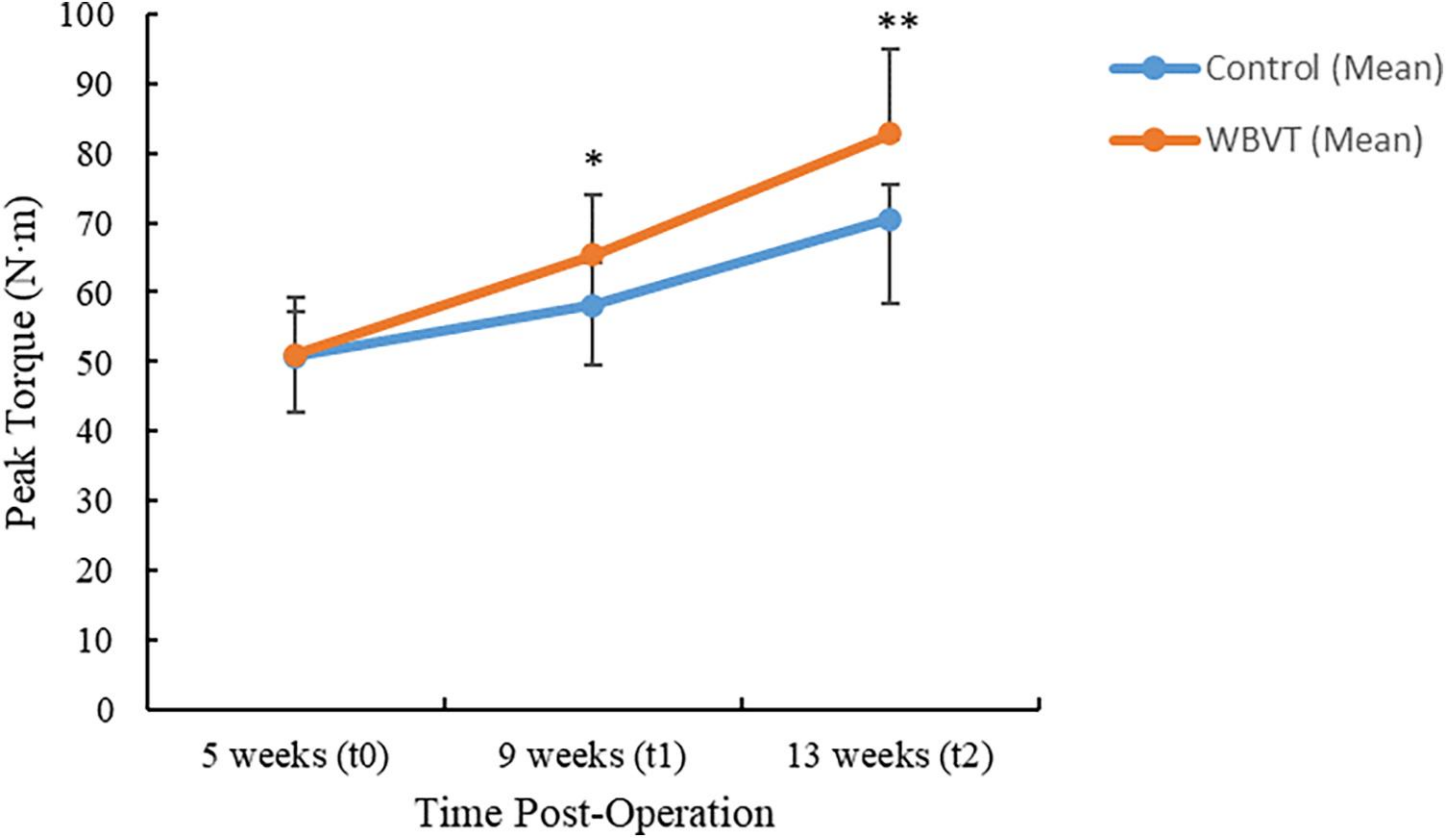
Changes in knee flexor peak torque over time. Data are presented as mean ± SD. * indicates a significant difference compared with the control group (*P* < 0.05), ** indicates a highly significant difference compared with the control group (*P* < 0.01).

Knee extensor PT demonstrated a significant Time × Group interaction [*F* (1.25, 32.50) = 7.378, *P* = 0.007, *η*^2^ = 0.221]. While both groups showed significant longitudinal improvements across all intervals (*P* < 0.001), simple effects analysis revealed that the WBVT group achieved significantly higher peak torque than the control group at both t1 and t2. No significant between-group difference was observed at baseline (t0, *P* = 0.899) (Figure 2).

#### 3.2.2 Knee flexor peak torque

**Figure 3:**
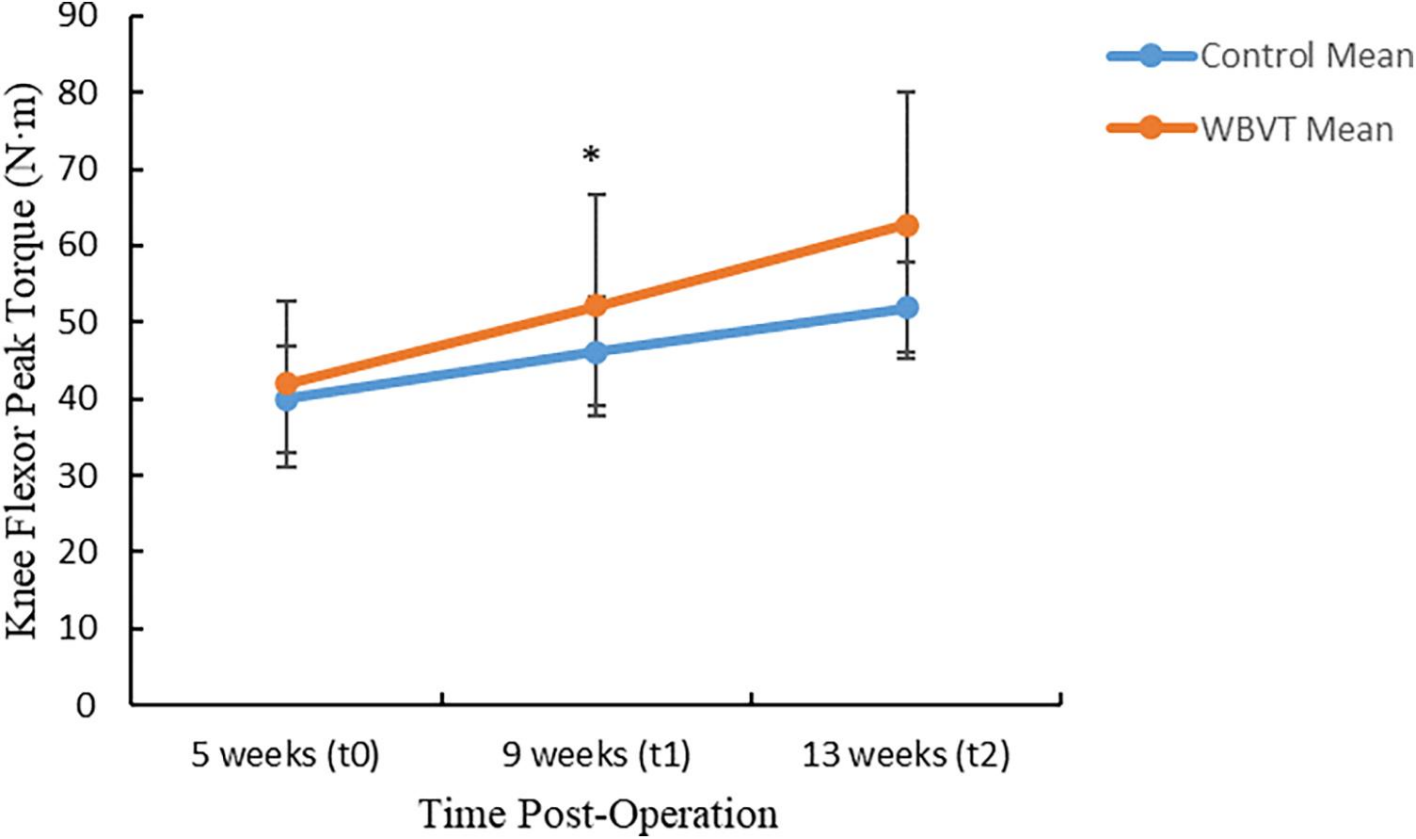
Changes in knee flexor peak torque over time. Data are presented as mean ± SD. * indicates a significant difference compared with the control group (*P* < 0.05).

Knee flexor PT demonstrated a significant Time × Group interaction [*F* (1.28, 33.38) = 4.114, *P* = 0.041, *η*^2^ = 0.137]. While both cohorts achieved progressive longitudinal gains from baseline through t2 (*P* < 0.05), simple effects analysis revealed that the WBVT group achieved significantly superior peak torque compared to the control group specifically at t2. No significant between-group differences were observed at t0 or t1 (*P* > 0.05) (Figure 3).

### 3.3 Proprioceptive function

#### 3.3.1 Joint position sense

##### 3.3.1.1 90°–60° joint position sense

**Figure 4:**
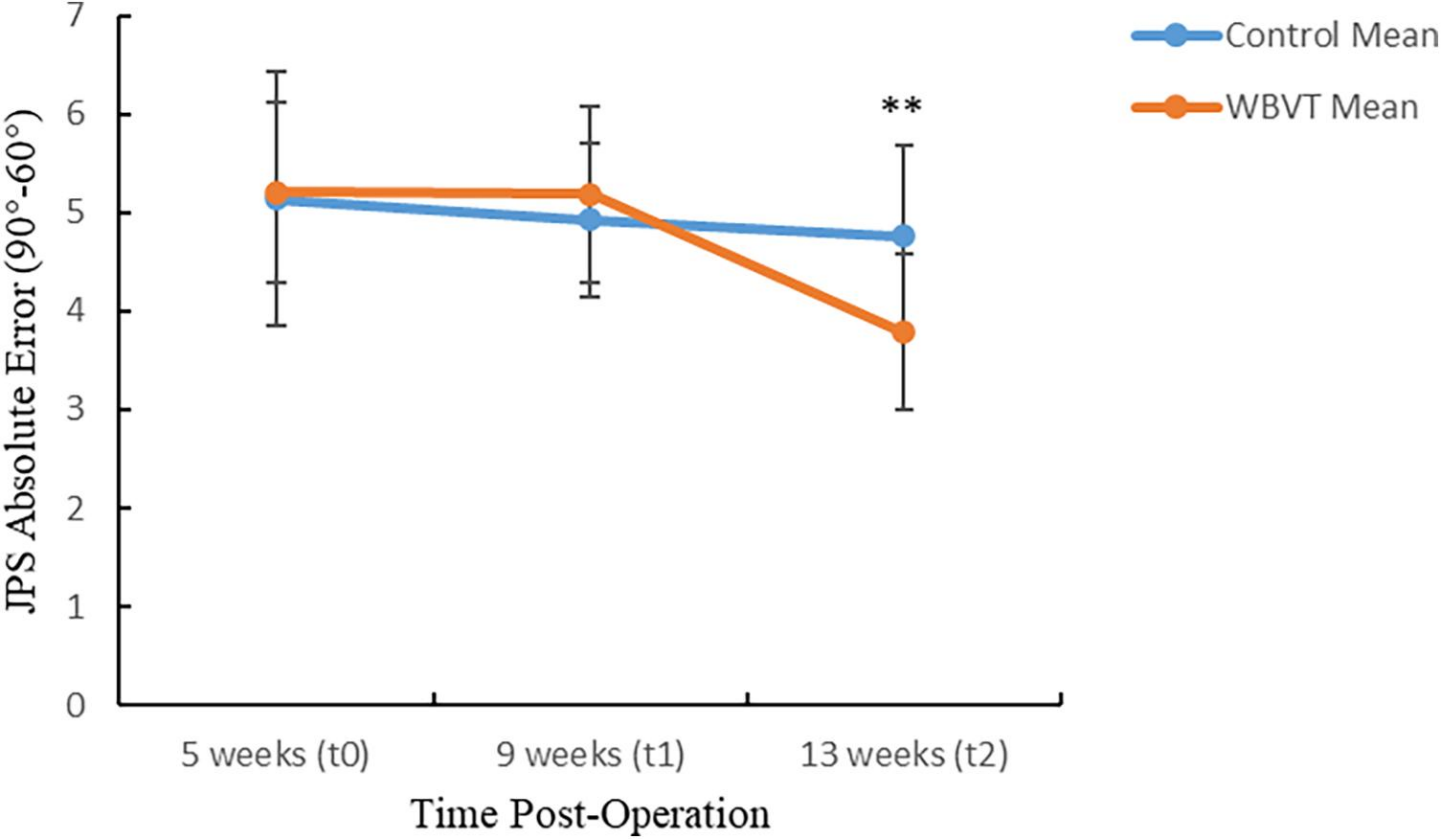
Comparison of joint position sense (90°–60°) absolute error between groups. Data are presented as mean ± SD. A lower value indicates better proprioception. ** indicates a highly significant difference compared with the control group (*P* < 0.01).

The 90°–60° JPS absolute error showed a significant Time × Group interaction [*F* (2, 52) *=* 3.416, *P* = 0.040, *η*^2^ = 0.116]. Simple effects analysis revealed that while the control group showed no significant changes over time (*P* = 0.677), the WBVT group demonstrated marked improvements by t2 compared to both baseline (t0: 5.21 ± 0.9° to t2: 3.79 ± 0.80°, *P* = 0.007) and t1 (*P* < 0.001). Consequently, the WBVT group achieved significantly better repositioning accuracy than the control group at t2 (*P* = 0.006), with no significant between-group differences observed at t0 or t1 (*P* > 0.05) (Figure 4).

##### 3.3.1.2 90°–30° joint position sense

**Figure 5:**
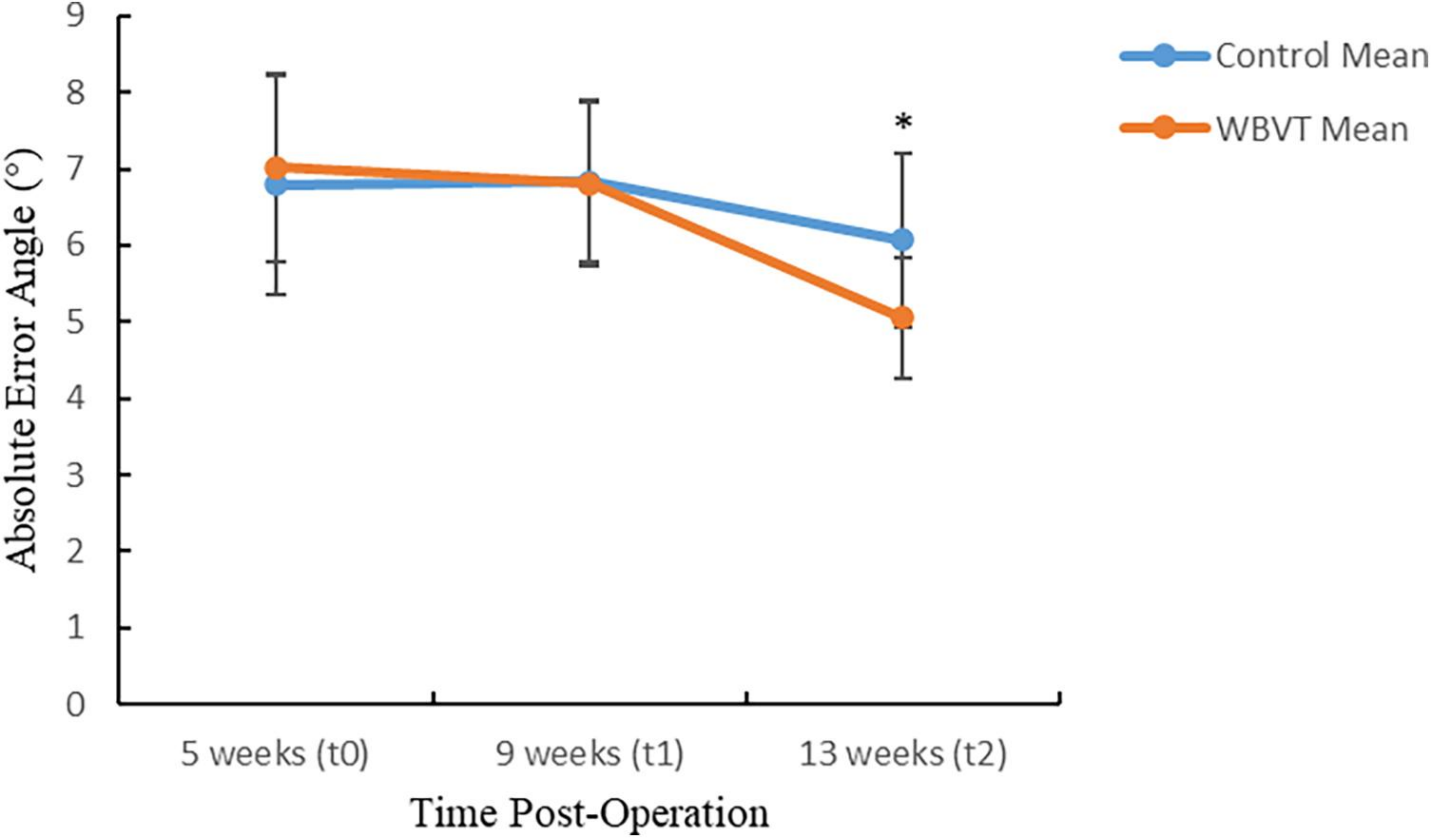
Comparison of joint position sense (90°–30°) absolute error. Data are expressed as mean ± SD. * indicates a significant difference compared with the control group (*P* < 0.05).

The 90°–30° JPS absolute error demonstrated a significant Time × Group interaction [*F* (2, 52) = 4.631, *P* = 0.014, *η*^2^ = 0.151]. Simple effects analysis revealed that the WBVT group achieved significantly better repositioning accuracy than the control group at the t2 (5.05 ± 0.80° vs. 6.07 ± 1.13°, *P* = 0.010). Within-group comparisons showed that the WBVT group improved significantly by t2 compared to both baseline (t0: 7.02 ± 1.24°, *P* < 0.001) and t1 (*P* < 0.001), whereas the control group did not differ significantly from baseline at the final follow-up (*P* > 0.05). No significant between-group differences were observed at t0 or t1 (*P* > 0.05) (Figure 5).

### 3.4 Joint kinesthesia

**Figure 6:**
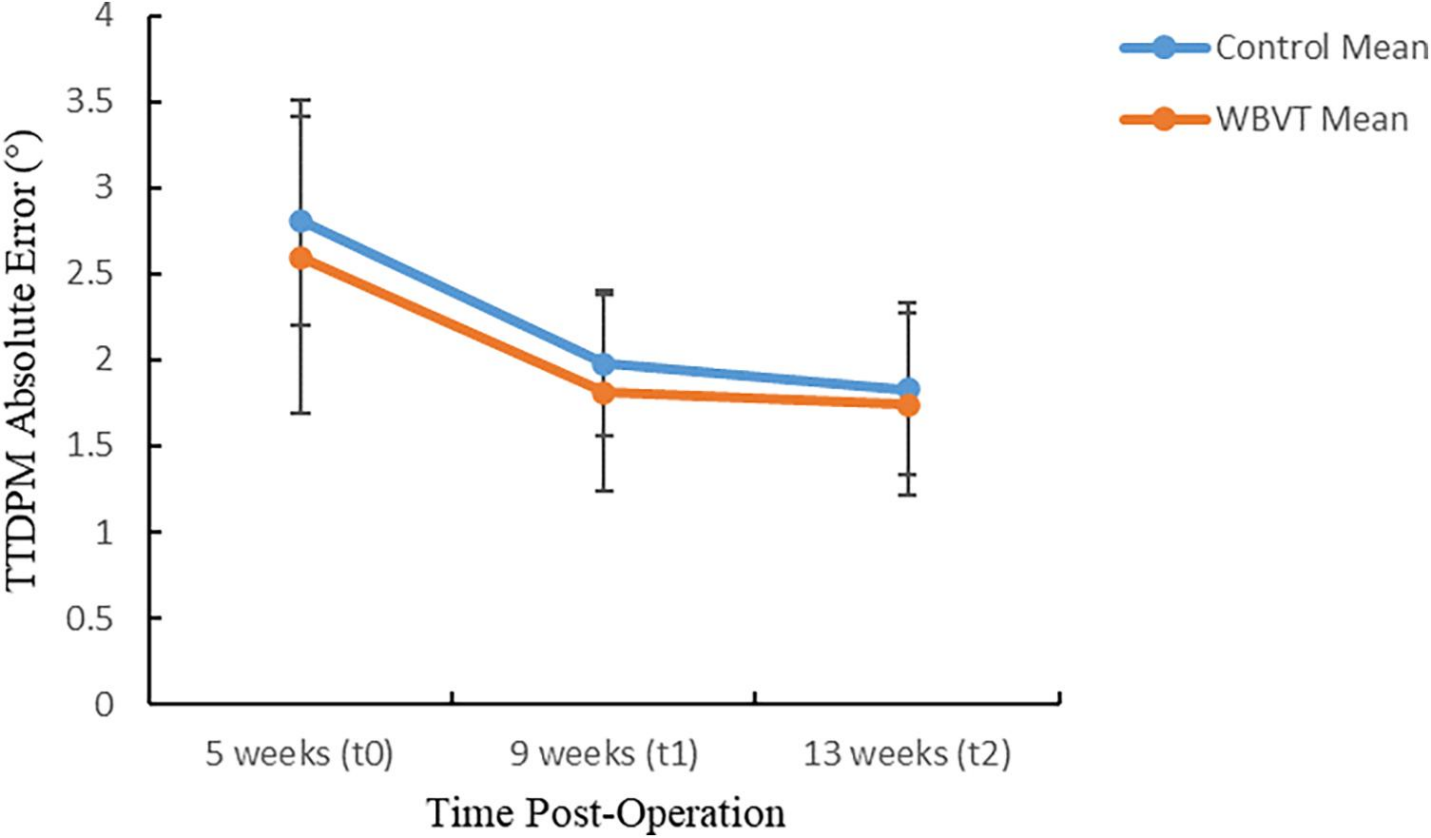
Comparison of TTDPM (joint kinesthesia) between groups. Data are presented as mean ± SD. Lower values indicate better kinesthesia. No significant differences were observed between the two groups at any time point (*P* > 0.05).

Analysis of the TTDPM absolute error revealed no significant Time × Group interaction [*F* (1.30, 33.70) = 0.161, *P* = 0.755, *η*^2^ = 0.006] and no significant main effect for Group (*P* = 0.423). However, a significant main effect for Time was observed [*F* (1.30, 33.70) = 45.118, *P* < 0.001, *η*^2^ = 0.634]. Both the WBVT and control groups showed marked improvements in kinesthetic sensitivity by t1 compared to baseline (t0, *P* < 0.001), with no further significant gains between t1 and t2 (*P* > 0.05). Throughout the study, no statistically significant differences existed between the two protocols at any time point (Figure 6).

### 3.5 Static balance

#### 3.5.1 Eyes-open balance

**Figure 7:**
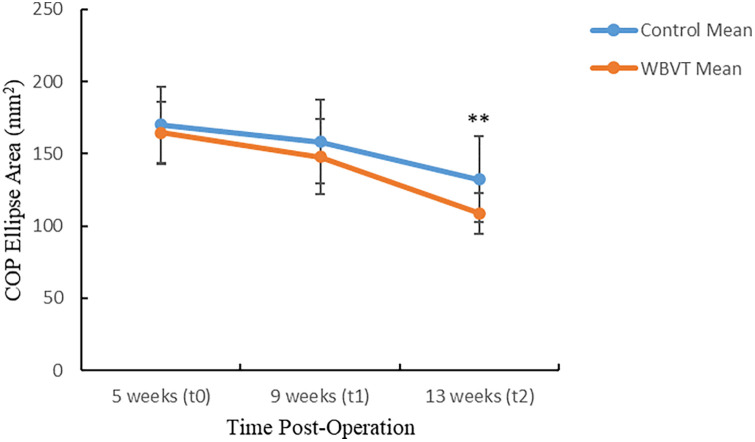
Comparison of COP ellipse area under eyes-open condition. Data are presented as mean ± SD. Lower values indicate better static balance. ** indicates a highly significant difference compared with the control group (*P* < 0.01).

The COP ellipse area under the eyes-open condition demonstrated a significant Time × Group interaction [*F* (1.63, 42.28) = 3.659, *P* = 0.043, *η*^2^ = 0.123]. Simple effects analysis revealed that the WBVT group achieved a significant reduction in postural sway by t1 (t1 vs. t0: 147.93 ± 25.97 vs. 164.57 ± 21.62 mm^2^, *P* < 0.001), whereas the control group did not show significant improvement until t2 (*P* < 0.001). Consequently, the WBVT group exhibited significantly superior static stability compared to the control group at t2 (108.79 ± 13.98 vs. 132.29 ± 29.87 mm^2^, *P* = 0.003), with no significant between-group differences observed at t0 or t1 (*P* > 0.05) (Figure 7).

#### 3.5.2 Eyes-closed balance

**Figure 8:**
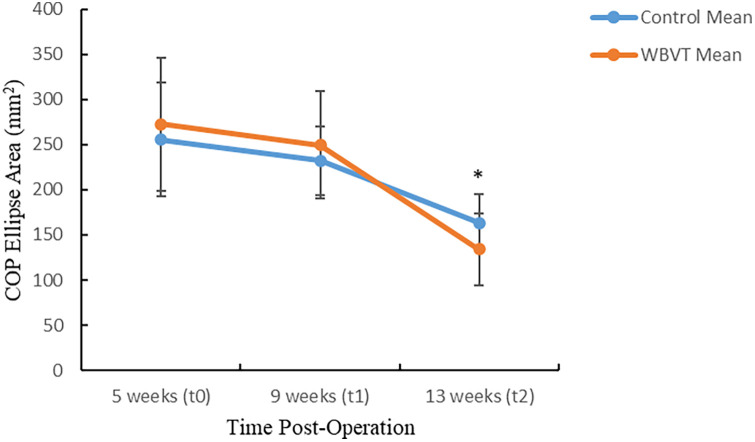
Comparison of COP ellipse area under eyes-closed condition. Data are presented as mean ± SD. Lower values indicate better static balance. * indicates a significant difference compared with the control group (*P* < 0.05).

The COP ellipse area under the eyes-closed condition demonstrated a significant Time × Group interaction [*F* (2, 52) = 4.185, *P* = 0.021, *η*^2^ = 0.139]. Simple effects analysis revealed that while neither group showed significant improvement at t1 (t1 vs. t0, *P* > 0.05), both cohorts achieved significant stability gains by t2 (*P* < 0.001). Crucially, the WBVT group exhibited significantly superior postural control compared to the control group at t2 (134.00 ± 39.69 vs. 163.50 ± 31.63 mm^2^, *P* = 0.039). No significant between-group differences were observed at t0 or t1 (*P* > 0.05) (Figure 8).

### 3.6 Lysholm knee score

**Figure 9:**
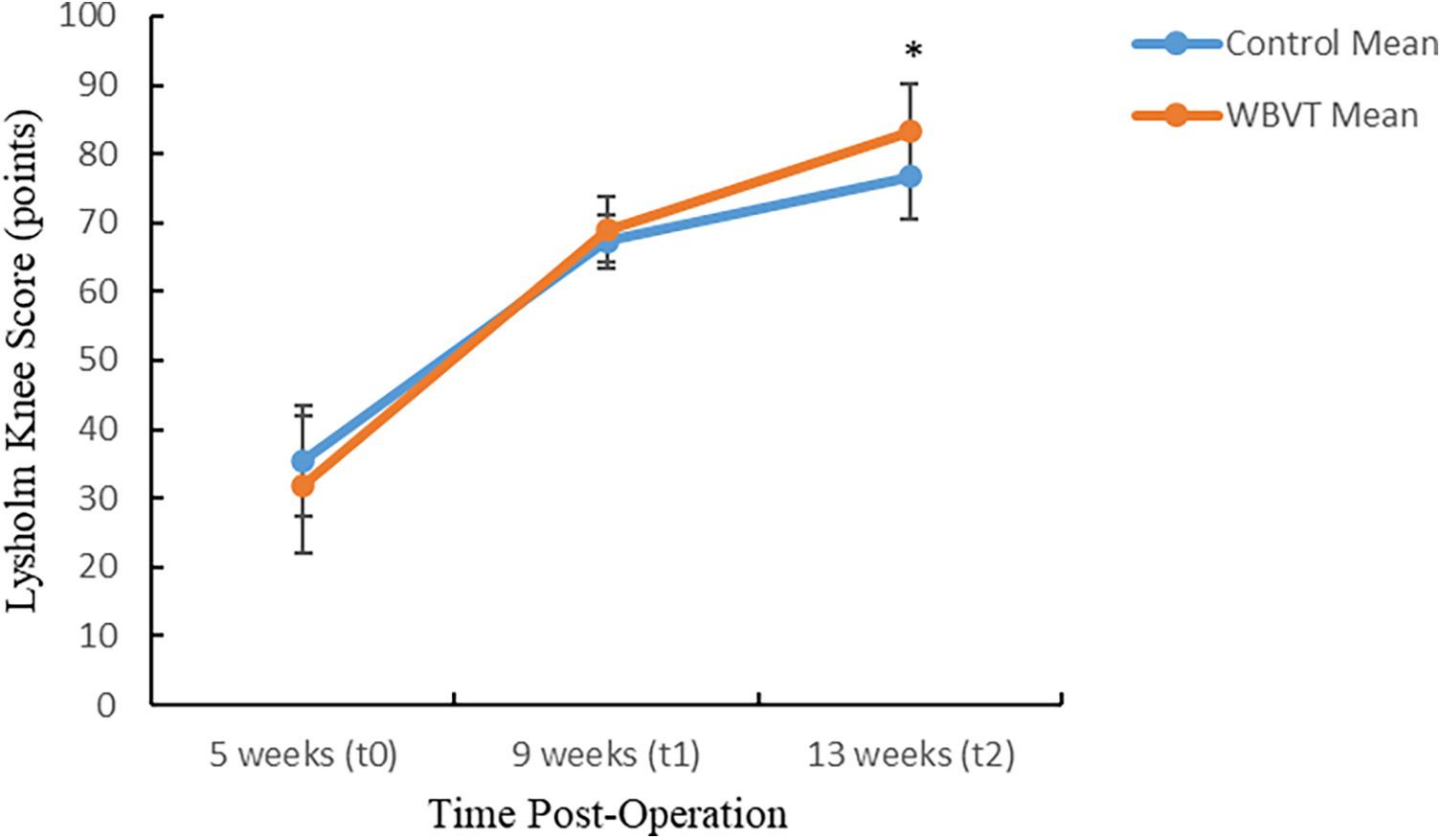
Comparison of lysholm knee scores between groups. Data are presented as mean ± SD. Higher scores indicate better knee function. * indicates a significant difference compared with the control group (*P* < 0.05).

The Lysholm knee score demonstrated a significant Time × Group interaction [*F* (1.62, 42.11) = 4.469, *P* = 0.024, *η*^2^ = 0.147]. While both groups exhibited continuous, significant functional improvements across all assessment intervals (*P* < 0.001), simple effects analysis revealed that the WBVT group achieved significantly higher subjective functional scores than the control group at t2 (83.29 ± 6.91 vs. 76.64 ± 6.13 points, *P* = 0.012). No significant between-group differences were observed at t0 or t1 (*P* > 0.05) (Figure 9).

### 3.7 Adverse events

No adverse events or complications related to the rehabilitation protocols or vibration interventions were reported in either group during the entire study period.

## 4 Discussion

The findings of the present study support our primary hypothesis. Our findings deliver a clear message: supplementing early ACLR rehabilitation with an 8-week SAV-WBVT protocol does more than just facilitate recovery—it significantly amplifies it. By initiating this intervention at the 5-week mark, we observed robust gains in peak torque, static balance, and joint position sense that clearly outpaced conventional care alone. What makes these results particularly compelling is the specificity of the benefit: SAV-WBVT appears to directly target the neuromuscular inhibition that typically creates a “ceiling” for early functional recovery. While we did not see a similar boost in kinesthesia, the marked improvements in subjective function and stability provide a strong rationale for integrating this modality into standard postoperative protocols.

### 4.1 Knee muscle strength recovery

Post-ACLR muscle deficits, driven by pain, immobilization, and neural inhibition, lead to quadriceps atrophy and impair daily living. Consequently, reactivating the quadriceps is a primary rehabilitation goal. Pamukoff et al. (13) demonstrated that a single WBVT session acutely improves quadriceps function and excitability. Furthermore, long-term WBVT interventions combined with conventional rehabilitation have been shown to enhance knee muscle strength (14).

Isokinetic testing (60°/s) was conducted at pre-intervention (t0), mid-intervention (t1), and post-intervention (t2) using a standard protocol. Recent studies confirm that early open-chain exercises do not increase graft laxity (15), particularly when knee flexion is maintained >30° (16, 17). Thus, testing was restricted to the 90°–30° range.

Both groups improved over time, likely due to subsiding postoperative inflammation. Joint effusion elevates intra-articular pressure, stimulating type II mechanoreceptors (18). These afferents, located in the ACL and capsule (19), inhibit quadriceps activation via Ib interneurons, affecting initial torque output (20). However, the WBVT group demonstrated superior recovery. This is attributed to the TVR, which enhances central nervous system adaptability (8). Biomechanically, vibration acts as an additional training load (21). Additionally, vibration-induced increases in circulation may enhance flexibility and torque output (22).

### 4.2 Proprioceptive recalibration

The ACL ensures knee stability via resident mechanoreceptors (23). Rupture compromises proprioception through receptor loss and altered neural input, while ACLR restores anatomy, mechanoreceptor reinnervation is delayed, perpetuating proprioceptive deficits (24). Assessment typically involves static JPS and dynamic TTDPM.

Due to methodological variations (active vs. passive modes), no standardized knee JPS protocol exists (25). Our findings parallel those of Zhao et al. (26), who reported magnified positional errors at 30° of knee flexion. Control subjects exhibited no longitudinal variation in the 90°–60° arc, yet achieved significant gains in the 90°–30° arc exclusively from mid- to post-intervention (t1–t2). The delayed proprioceptive improvement during the initial phase (t0–t1) likely stems from postoperative inflammatory responses.

By week 13, the WBVT group demonstrated significant JPS gains compared to controls and baseline. Although earlier investigations reported an absence of proprioceptive adaptations following similar protocols (27), the divergent outcomes in the present study are likely driven by methodological variations in testing. Specifically, whereas previous research relied on passive-passive reproduction, the passive-active protocol employed here deliberately recruits muscle spindles to amplify sensory afference (28). Nevertheless, the clinical utility of these statistical gains remains questionable. The absolute error reductions were confined to <2°, failing to meet the established 5° threshold required for meaningful clinical impact (29). Furthermore, because comprehensive mechanoreceptor reintegration typically requires more than six months, and dynamic stability relies on complex multi-joint coordination (26, 30), these early sensory adaptations should be viewed strictly as preliminary data.

Analysis of kinesthesia (TTDPM) revealed equivalent early improvements (t0–t1) in both cohorts, with no subsequent gains or group interactions. Contextualizing these findings is challenging due to the scarcity of post-ACLR TTDPM literature; notably, a recent meta-analysis (31) observed no substantial kinesthetic deficits relative to healthy populations. Furthermore, documented bilateral sensorimotor impairments following unilateral ACL injury precluded the use of the uninjured limb as a reliable internal control (26). Consequently, the additive benefit of WBVT on kinesthetic recovery remains indeterminate.

### 4.3 Static balance and postural control

The maintenance of postural stability necessitates the continuous integration of visual, vestibular, and proprioceptive afferents (32). Because this regulatory process is predominantly governed by unconscious proprioception, peripheral sensorimotor deficits fundamentally compromise stance stability, manifesting as exacerbated postural sway. Although ACLR-induced deficits theoretically compromise balance, findings regarding static deficits—particularly during single-leg stance—remain inconsistent (33). This inconsistency likely stems from a learning effect, as rehabilitation often emphasizes single-leg stability, potentially masking deficits relative to controls.

Consequently, double-leg stance is advocated as a reliable metric (34). Gokalp et al. (35) reported that postural instability peaks at 4 weeks post-ACLR and normalizes by weeks 8–12. In the present study, using double-leg stance, the WBVT group demonstrated significant reductions in the COP ellipse area under eyes-open conditions earlier than the control group, which only showed improvement relative to baseline at post-intervention (t2). Under eyes-closed conditions, neither group improved at mid-intervention (t1); however, after 8 weeks of intervention, the WBVT group exhibited significantly superior stability compared to controls.

These findings align with previous trials (36–38), which consistently report that WBVT enhances balance regardless of initiation timing. Notably, Moez (37) and Berschin et al. (38) demonstrated that standalone WBVT protocols could yield superior sensorimotor outcomes compared to conventional strength and proprioceptive training.

Mechanistically, these improvements are attributed to enhanced motor unit synchronization and the restoration of joint position sense. Uniquely, side-alternating WBVT generates differential vertical displacement, inducing rapid, alternating loading of the lower limbs. This mechanical oscillation necessitates compensatory core muscle activation (39), further optimizing postural stability.

### 4.4 Patient-reported functional outcomes

The Lysholm Knee Score is a standard metric for assessing post-ACLR functional status. Previous studies have confirmed that the Chinese translated version possesses robust measurement stability and accuracy for use in Chinese patients (40). In this study, both groups exhibited significant longitudinal improvements after 4 and 8 weeks of intervention. However, between-group analysis revealed that the WBVT group achieved significantly higher scores than the control group only after 8 weeks.

The universal improvement observed at 4 weeks is likely attributable to the resolution of acute postoperative pain and swelling, which facilitates basic tasks like stair climbing. Conversely, the superior outcomes in the WBVT group at 8 weeks were driven by reduced reports of subjective instability and locking, alongside enhanced squatting performance. These functional gains mirror the observed improvements in proprioception and isokinetic strength, corroborating the correlation between Lysholm scores and knee torque deficits reported by Andrade et al. (41).

## 5 Conclusion

The incorporation of an 8-week SAV-WBVT protocol, initiated 5 weeks post-ACLR, significantly accelerates short-term functional recovery. Compared to conventional rehabilitation alone, it provides superior clinical improvements in isokinetic muscle strength of the knee extensors and flexors, joint position sense, static balance, and patient-reported knee function. However, it offers no additional therapeutic benefit for joint kinesthesia.

## 6 Limitations

We acknowledge several limitations. First, the exclusive recruitment of males restricts generalizability to female populations. Second, uncontrolled preoperative variables, such as injury-to-surgery intervals, introduced some clinical heterogeneity. Third, our reliance on per-protocol analysis may carry attrition bias relative to an intention-to-treat approach. Fourth, the perceptible nature of vibration precluded blinding—an inherent constraint in rehabilitation trials—though standardized protocols were used to minimize performance bias. Fifth, balance assessment was confined to static measures to prioritize early-phase graft safety. Finally, the 13-week follow-up is insufficient to predict long-term return-to-sport readiness.

## Data Availability

The raw data supporting the conclusions of this article will be made available by the authors, without undue reservation.
